# Expansion of pneumococcal serotype 23F and 14 lineages with genotypic changes in capsule polysaccharide locus and virulence gene profiles post introduction of pneumococcal conjugate vaccine in Blantyre, Malawi

**DOI:** 10.1099/mgen.0.001264

**Published:** 2024-06-19

**Authors:** Rory Cave, Akuzike Kalizang'oma, Chrispin Chaguza, Thandie S. Mwalukomo, Arox Kamng’ona, Comfort Brown, Jacquline Msefula, Farouck Bonomali, Roseline Nyirenda, Todd D. Swarthout, Brenda Kwambana-Adams, Neil French, Robert S. Heyderman

**Affiliations:** 1 Mucosal Pathogens Research Group, Research Department of Infection, Division of Infection & Immunity, University College London, London, UK; 2 Malawi Liverpool Wellcome Programme, Blantyre, Malawi; 3 Parasites and Microbes, Wellcome Sanger Institute, Cambridge, UK; 4 Department of Epidemiology of Microbial Diseases, Yale School of Public Health, Yale University, New Haven, CT, USA; 5 Kamuzu University of Health Sciences, Blantyre, Malawi; 6 Julius Center for Health Sciences and Primary Care, University Medical Centre Utrecht, Utrecht, Netherlands; 7 Clinical Infection, Microbiology and Immunology, Institute of Infection Veterinary & Ecological Science, University of Liverpool, Liverpool, UK

**Keywords:** antimicrobial resistance, Africa, Capsule polysaccharide *locus*, PCV13, *Streptococcus pneumoniae*, vaccine escape

## Abstract

Since the introduction of the 13-valent pneumococcal conjugate vaccine (PCV13) in Malawi in 2011, there has been persistent carriage of vaccine serotype (VT) *Streptococcus pneumoniae*, despite high vaccine coverage. To determine if there has been a genetic change within the VT capsule polysaccharide (cps) loci since the vaccine’s introduction, we compared 1022 whole-genome-sequenced VT isolates from 1998 to 2019. We identified the clonal expansion of a multidrug-resistant, penicillin non-susceptible serotype 23F GPSC14-ST2059 lineage, a serotype 14 GPSC9-ST782 lineage and a novel serotype 14 sequence type GPSC9-ST18728 lineage. Serotype 23F GPSC14-ST2059 had an I253T mutation within the capsule oligosaccharide repeat unit polymerase Wzy protein, which is predicted *in silico* to alter the protein pocket cavity. Moreover, serotype 23F GPSC14-ST2059 had SNPs in the DNA binding sites for the cps transcriptional repressors CspR and SpxR. Serotype 14 GPSC9-ST782 harbours a non-truncated version of the large repetitive protein (Lrp), containing a Cna protein B-type domain which is also present in proteins associated with infection and colonisation. These emergent lineages also harboured genes associated with antibiotic resistance, and the promotion of colonisation and infection which were absent in other lineages of the same serotype. Together these data suggest that in addition to serotype replacement, modifications of the capsule locus associated with changes in virulence factor expression and antibiotic resistance may promote vaccine escape. In summary, the study highlights that the persistence of vaccine serotype carriage despite high vaccine coverage in Malawi may be partly caused by expansion of VT lineages post-PCV13 rollout.

**Impact Statement**

Our findings highlight the potential for clonal expansion of multidrug-resistant, penicillin-non-susceptible vaccine serotype lineages with capsule locus modifications, within a high carriage and disease burden population. This shift has occurred among young children where there has been high vaccine coverage, posing challenges for effective vaccine scheduling and design. Furthermore, this study emphasises the importance of ongoing *Streptococcus pneumoniae* genomic surveillance as new or modified pneumococcal vaccines are implemented.

## Data Summary

Whole genome sequencing assemblies for the PCVPA survey have been deposited in the BioProject PRJNA1011974.

## Introduction

Pneumonia, meningitis and sepsis caused by *Streptococcus pneumoniae* (the pneumococcus) is a major global public health concern, with an estimated 300000 global deaths due to invasive disease reported among children aged under 5 of which 57% occur within resource poor settings [1,2]. Asymptomatic nasopharyngeal carriage of the pneumococcus is a prerequisite for pneumococcal disease, transmission, and the development of natural immunity [3]. Since the introduction of pneumococcal conjugate vaccines (PCVs) in childhood vaccination programmes worldwide, there has been a considerable reduction in invasive disease [4].

The outer capsule polysaccharide (cps) is a major pneumococcal virulence factor, protecting the pneumococcus against desiccation, complement-mediated opsonophagocytosis and other host antimicrobial pathways [5,7]. The cps biosynthesis genes are found on a single locus controlled by a single promoter region for most serotypes [8]. Cps consists of diverse sugar structures that vary among isolates, serving as the basis for classifying *S. pneumoniae* serotypes, with more than 100 immunologically-distinct serotypes identified to-date [9]. PCVs are formulated with a select array of serotype-specific capsule polysaccharides, chosen to target the most commonly occurring invasive serotypes, with a particular focus on those that cause the most severe diseases or are associated with AMR [10].

Since the 2011 PCV13 rollout in Blantyre, Malawi, it has been shown that there was a reduction in vaccine serotype (VT) invasive pneumococcal disease (IPD) with the incidence of post-PCV13 VT IPD 74% lower among children aged 1–4 years, and 79% lower among children aged 5–14 years from 2006 to 2018 [11]. However, among PCV13 age ineligible populations, it was noted to be only 38% lower VT IPD among infants and 47% lower among adolescents and adults. We have also shown that VT IPD has persisted amongst infants <90 days old [12]. Alongside this, it has been shown that in contrast to high income settings, there is considerable residual VT carriage, 7 years after PCV13 introduction despite high vaccine uptake [13]. We have also shown that this imperfect direct and indirect control of pneumococcal carriage and disease is associated with waning of protective vaccine-induced anti-pneumococcal immunity in the first year of life [14].

The pneumococcus is highly transformable such that the *cps* locus, a known recombination hotspot, often acquires changes and can facilitate pneumococcal vaccine escape [15]. Serotype switching from VT to non-vaccine serotypes (NVT), gene deletions or mutations resulting in pseudogenes lead to capsule loss or the formation of capsule types that alter the biochemical properties of the VT capsule, giving them serological properties distinct from the previous capsule type [16,19]. However, genetic changes within the cps locus which do not lead to capsule switch loss, or altered capsule serotype could also enable VT serotype persistence post-PCV rollout [20]. Here, we investigated the hypothesis that the residual VT IPD and persistent VT carriage observed following PCV13 introduction in Malawi, is at least in part due to the clonal expansion of VT lineages that have acquired changes in their *cps* locus while maintaining their serotype. We further postulate that these capsule locus variants have also acquired genetic traits that together could promote a competitive advantage in colonisation and transmission, with the potential to result in vaccine escape.

## Methods

### Whole genome sequences

We obtained the whole genome draft assemblies of PCV13 VT *S. pneumoniae* isolates from Blantyre, Malawi, collected between 1998 and 2015 through the Global Pneumococcal Sequencing Project (GPS) dataset, a collection of international genomes from carriage and disease [21]. This dataset was originally used to understand the structure of the pneumococcal population and the impact of the PCV vaccine (Table S1, available in the online version of this article). For the isolates from Blantyre, the carriage isolates (*n*=51) from the GPS dataset were part of the VacSurv Pneumonia study of children under the age of three, a survey conducted to assess PCV13 effectiveness between 2013 and 2015 in Blantyre, Malawi. The disease isolates (*n*=430) were from routine sureveillance at the Queen Elizabeth Central Hospital, Blantyre, Malawi, for laboratory-confirmed invasive pneumococcal disease (bacteraemia or meningitis). These included all age groups with known isolation dates between 1998 and 2015 [11,22,25]. We also used the Pneumococcal Conjugate Vaccine Prospective Analysis (PCVPA) dataset (*n*=734), consisting of serologically typed carriage isolates collected from vaccinated children aged 2 to 7 years (*n*=436), unvaccinated children aged 5 to 10 years (*n*=220), and adults aged 18 to 40 years (*n*=81) living with HIV between 2015 and 2019 [13,26]. From the PCVPA dataset, each year 600 swabs were taken from vaccinated children and 400 swabs from unvaccinated children and 400 swabs from HIV-positive adults except for 2019 where only half the number of swabs was taken for each group. Additionally, for the purpose of genomic comparison between Blantyre VT isolates with those isolated in other countries we incorporated publicly available isolates from Pathogenwatch which contains sequences from carriage and disease from early 1900s to 2021 (https://pathogen.watch/, accessed February 2023).

### Genome genetic typing and annotation

Genetic typing and antimicrobial resistance genotype of isolates was also conducted using Pathogenwatch. Lineages were defined by Pathogenwatch using the Global Pneumococcal Sequence Cluster (GPSC) nomenclature employing the PopPUNK framework, and the Multi-Locus Sequence Type (MLST) system based on the pneumococcal scheme [27,29]. Full genome annotation was conducted using Bakta v1.9.1 [30].

### *cps* locus comparison

We used parsnp v1.7.4 (https://github.com/marbl/parsnp) to extract and identify single nucleotide polymorphisms (SNPs) within the Blantyre pneumococcal *cps* locus by aligning them against reference serotype specific *cps* locus described by Bentley *et al*. [8]. These SNPs were then annotated to distinguish synonymous from non-synonymous mutations using the vcf-annotator tool v0.5 (https://github.com/rpetit3/vcf-annotator). A phylogenetic tree from the alignment of the *cps* locus was constructed using IQ-TREE v2.1.2 with the best model for each alignment selected by ModelFinder [31,32]. Phylogenetic trees and SNPs within the *cps* locus were visualised using the R package ggtree v3.18 [33]. BLASTP v2.14.1 was used to determine if similar mutations were found in the amino acid sequence of isolates from other countries [34].

We performed sequence alignment for the *cps* locus using blastn v2.14.1 to enhance the detection of indels within the *cps* locus that are segmented into multiple contigs. The alignment coverage against the reference was then visualised using the R package gggenomes v0.9.12 (https://github.com/thackl/gggenomes).

To determine genetic synteny within the intergenic regions of the *cps* locus, we extracted the DNA sequences located between *dexB* and *wzg* for each individual genome. Synteny was established by aligning these sequences with one another using minimap2, and the resulting synteny patterns were visualised using the R package gggenomes [35]. We investigated alterations within the 37-CE region located upstream of the cps promoter, where the transcriptional factors SpxR and CpsR are known to bind to suppress expression of the cps locus [36]. To locate and extract the 37-CE sequence, we developed a custom Python script (https://github.com/rorycave/37-CE_finder) that identified the position of the sequence pattern ‘TTGAAAC,’ which is typically conserved in the 37-CE region across various serotypes within the *cps* locus intergenic region. Subsequently, the 37-CE sequences were extracted using bedtools v2.28 'getfasta' commands based on their sequence positions, including 156 nucleotides upstream and 14 nucleotides downstream of the sequence [37]. The extracted sequences were then manually checked by aligning them to the reference 37-CE sequence using Clustal Omega v1.2.4 [36,38].

### The impact of non-synonymous SNPs on protein structure within the *cps* locus

To determine the impact of nonsynonymous SNPs on a protein structure within the *cps* locus, we first used SWISS-MODEL, to find homologous protein models that had a Global Model Quality Estimate (GMQE) >0.95 [39]. Furthermore, within that GMQE range we chose X-ray diffraction models if present over models predicted by *in silico* methods. For transmembrane proteins that only have *in silico* predicted models, TMBed was used to identify if mutations occurred in the transmembrane cytoplasmic or extracellular domain of the protein [40]. Additionally, we used PrankWeb 3 web server which runs P2RANK to determine if the mutation occurred in a protein pocket, and Missesne3D to assess whether mutations would cause structural damage to the protein’s 3D structure [41,43].

### Phylogenetic, lineage expansion and accessory genome analysis

A core SNP maximum-likelihood (ML) phylogenetic tree was constructed for each serotype: one comprising Malawian isolates only, and one comprising all global isolates within same lineage. This was done by aligning assemblies to a lineage specific complete reference genome sequence using Snippy v4.6.0 (https://github.com/tseemann/snippy). Recombination within aligned sequences was then filtered out with Gubbins v3.3.1 [44]. A phylogenetic tree was then constructed from recombinant-free alignment using IQ-TREE v2.1.2 with the best model for each alignment using selected by ModelFinder and set ultrafast bootstrap replication to 1000 [31,32]. The phylogenetic tree was visualised and annotated in Microreact [45].

To infer changes in the effective population size over time and clonal expansion events of a lineage, a dated phylogenetic tree of the Malawian isolates was first constructed using the R package BactDating v1.1.2 with the mixedcarc model and setting the MCMC to 5×10⁷ iterations. Clonal expansion and effective population size were inferred using the R package CaveDive v0.1.1, applying previously set priors from Helekal *et al*. to infer pneumococcal lineage expansion with the MCMC set to 1×10⁸ [46].

To find differences in the accessory genome between lineages that have the same serotype, a pangenome from the annotated genome for each serotype was then constructed using Panaroo v1.3.4 with the merge paralogs setting [47]. Scoary v1.6.16 was then used to identify genes that belong to certain lineages that were absent in others [48]. A reference free core-genome phylogenetic tree without recombination removed for each serotype was produce from Panaroo alignment using IQ-TREE v2.1.2 as previously stated to compare accessory gene content between lineages.

### Statistical analysis

To assess changes in genotype carriage over time for vaccine serotypes, we used the Chi-squared test for trend in proportions using the R stats package. The denominator was the number of swabs taken each year.

## Results

### Expansion of *cps* locus variant lineages of serotype 23F and 14 following PCV13 introduction

To determine if the predominant VT cps locus genotypes changed after PCV13 introduction in Malawi, we compared the PCVPA dataset (2015 to 2019) to earlier pneumococcal sequences (1998 to 2014) in Blantyre, Malawi. Serotype 23F and 14 lineages were found to have expanded with changes in their *cps* locus genotypes.

**Fig. 1.**
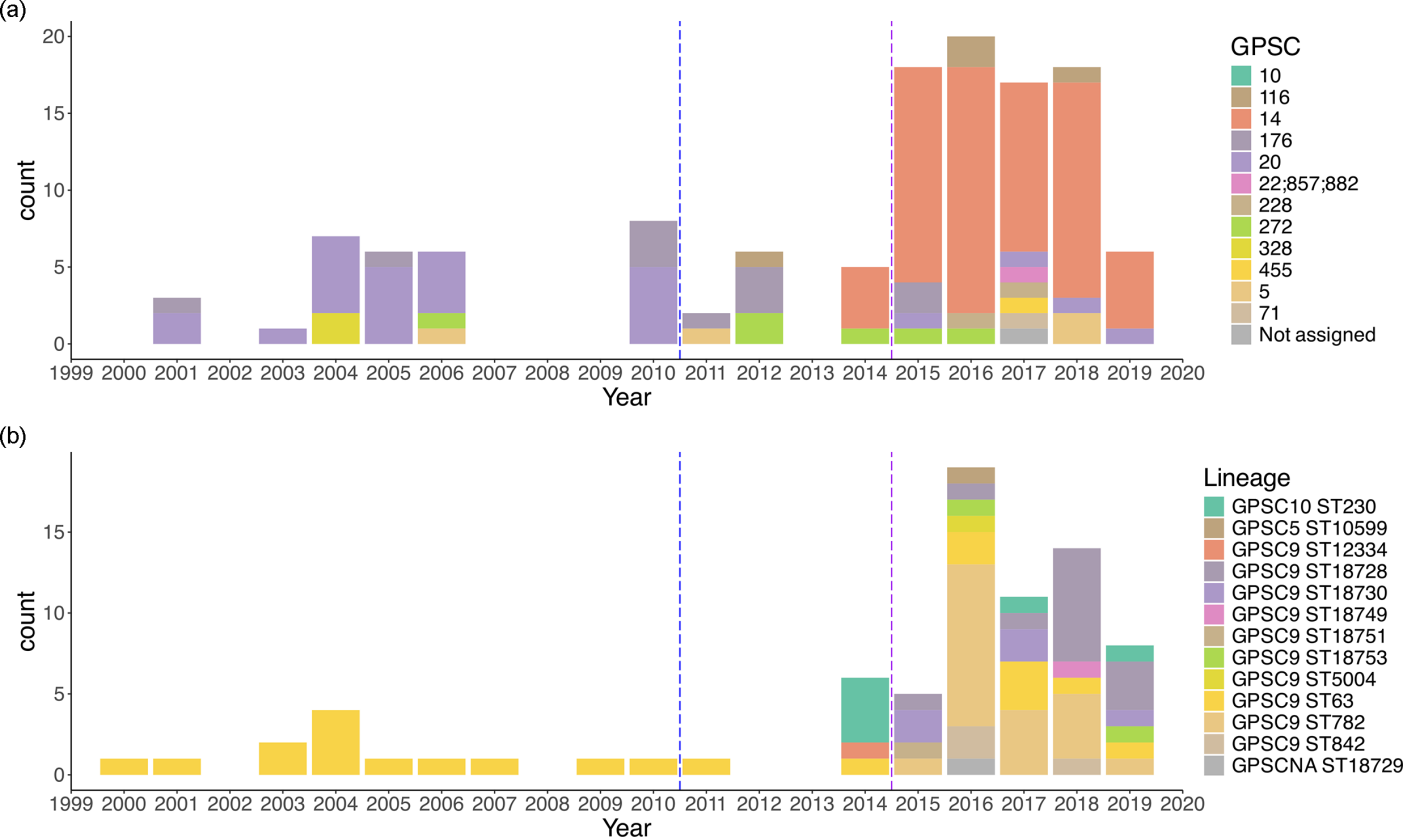
Change in genetic lineage/strain overtime among A) Serotype 23F and B) Serotype 14 isolates. Blue dashed line indicates the time that PCV13 was introduced in Blantyre, Malawi. Purple line indicates the start of PCVPA survey.

For serotype 23F isolates (*n*=125, collected between 1999 and 2019), the dominant lineage shifted from GPSC20 pre-PCV13 rollout (*n*=23, 69.7% of the serotype 23F isolates pre-PCV13) to a GPSC14 lineage that was dominant from 2014 onwards (*n*=64, 76.1% of the serotype 23F isolates after 2014) (Fig. 1a). This includes the emergence of GPSC14 ST2059 (*n*=48, 57.1% of the total serotype 23F isolates after 2014), which became dominant among all 23F serotype genotypes collected in the PCVPA dataset. It was present in vaccinated children (*n*=25, 55.6% of the total serotype 23F isolates among vaccinated children), unvaccinated children (*n*=14, 58.3% of the total serotype 23F isolates among unvaccinated children), and HIV-positive adults (*n*=3, 75% of the total serotype 23F isolates among HIV-positive adults). This indicates that these 23F strains were shared among different population groups in Blantyre. The emergence of GPSC14 ST2059 was predicted to be a clonal expansion (82% chance) of the GPSC14 lineage, based on a posterior inference under the CaveDive model from a dated phylogenetic tree by aligning against the complete genome of a GPSC14 *S. pneumoniae* isolate (GenBank accession: NZ_LR216043) (Fig. S1a). From the prevalence of GPSC14 isolates among pneumococcal carriers in the PCVPA study, we observed no significant change over time (*P*=0.15), indicating that the lineage is persisting in the population during the PCVPA study (Fig. S2). However, from the expansion of branch eight of the CaveDive model, which contains the ST2059 isolates, we observed an increase in the effective population size starting 8 years prior to the most recent isolate date (2019) (Fig. S1b). This timeframe coincides with the introduction of the PCV13 vaccine in Malawi. We propose that the GPSC14 ST2059 isolates might have developed capabilities that enabled escape from PCV-induced immunity [49].

For serotype 14 isolates (*n*=77, collected between 2000 and 2019), the dominant GPSC9 remained unchanged after the introduction of PCV13 (Fig. 1b). However, there was a shift in the most dominant STs, transitioning from GPSC9 ST63 pre-vaccine (*n*=13, 100% of the serotype 14 pre-vaccine) to GPSC9 ST782 (*n*=20, 35.1% of PCVPA serotype 14 isolates) between 2015 and 2019. We also observed the clonal expansion of a novel serotype 14 sequence type, ST18728 (*n*=13, 26.3% of PCVPA serotype 14 isolates), a single locus variant (SLV) of ST782. Isolates were shown to be phylogenetically closely related to those of the ST782 isolates, which were dominant from 2018 to 2019, coinciding with a reduction in the prevalence of ST882 in the PCVPA dataset (Figs S2 and S3). This expansion was not detected by the CaveDive model from a dated phylogenetic tree by aligning against the complete genome of GPSC9 *S. pneumoniae* isolate (GenBank accession: NZ_LR216043) (Fig. S3). This was most likely due to the very recent expansion and the low sample size of these sequence types [50]. Moreover, there was no significant change in the overall for ST782 (*P*=0.12) but there was a significant increase in ST18728 (*P*=0.013) during the PCVPA study (Fig. S2). Additionally, the same predomint lineages were found in the PCVPA dataset for serotype 14 for both vaccinated (ST782, *n*=15, 33.4% and ST18728, *n*=10, 22.3% of the total PCVPA serotype 14 isolates from vaccinated children) and unvaccinated children (ST782, *n*=5, 45.5% and ST18728, *n*=3, 27.3% of the total serotype 14 PCVPA isolates from unvaccinated children), but not for HIV-positive adults; however, only a single serotype 14 isolate was recovered in the PCVPA study for this group (GPSC10 ST230).

### Genomic changes within 23F GPSC14 *cps* locus predicted to impact on capsule expression and phenotype

**Fig. 2.**
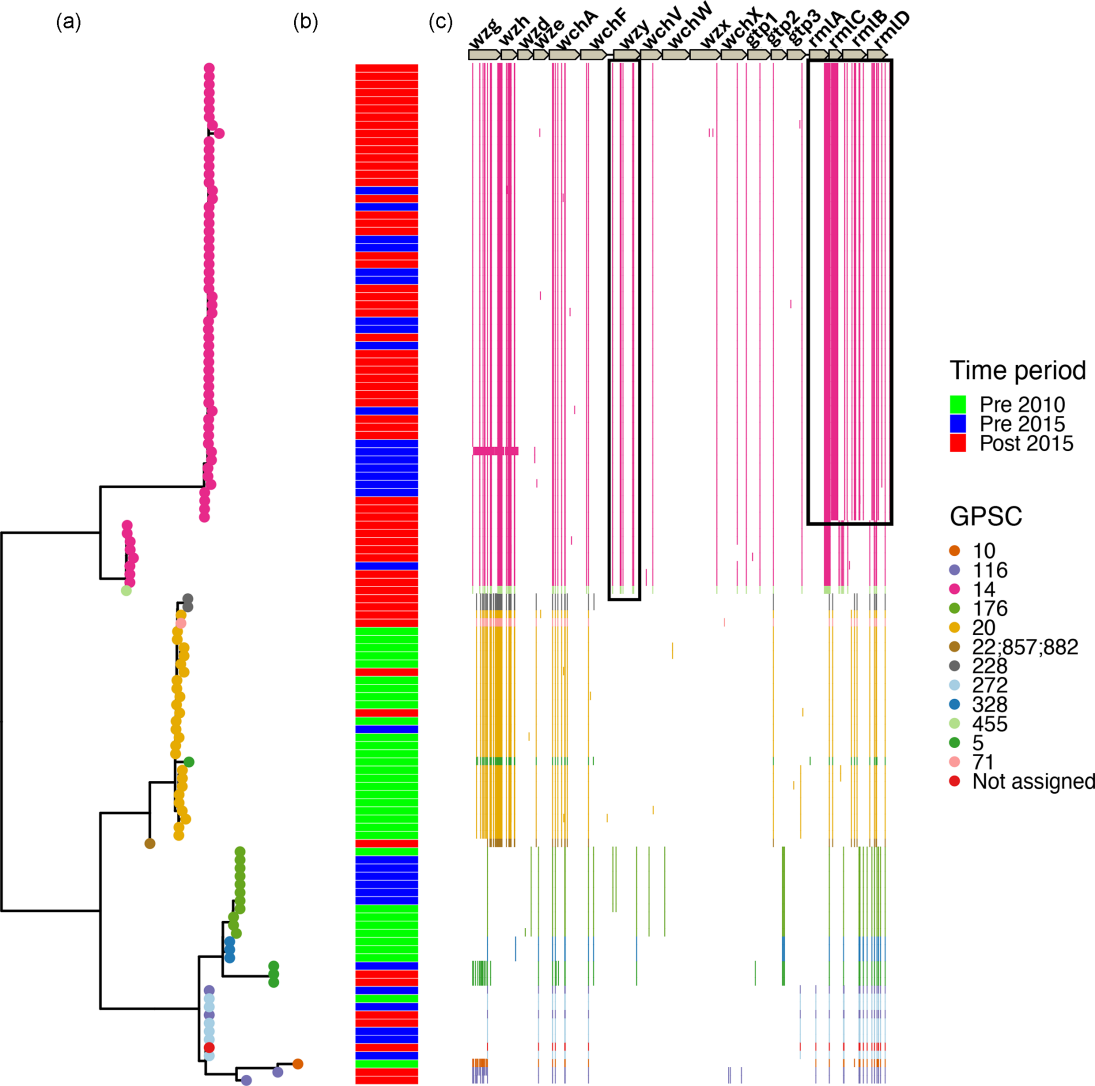
Maximum likelihood phylogenetic tree and position of SNPs within serotype 23F *cps* locus shows that change in genotype in *wzy* and *rmlACBD* in the emergent GPSC14 *S. pneumoniae* isolates in Blantyre, Malawi. (a) Phylogenetic tree of serotype 23F *cps* locus with tips on tree representing isolates GPSC. (b) Colour tiles represent the time period isolates were sampled from. (c) Colour lines represent position of SNPs within *cps* locus for each isolate and their colour represents their GPSC.

To explore the hypothesis that the clonal expansion of 23F GPSC14 ST2059 post-PCV13 introduction and the decline in 23F GPSC20 may have been due to a competitive advantage, we first looked for genetic differences within the *cps* locus. The alignment with the reference 23F *cps* locus (GenBank accession: CR931685) revealed differences in the 23F GPSC14 lineage SNP patterns compared to the 23F GPSC20 lineages, with the most significant SNPs in the 23F GPSC14 lineages being four non-synonymous SNPs, resulting in amino acid changes: W54S, L56F, T237I, and I253T, in the oligosaccharide repeat unit polymerase gene, *wzy*. These same mutations were also found in a single serotype 23F GPSC455 isolate (Fig. 2).

Additionally, from the *cps* locus alignment, we identified a subpopulation within the GPSC14 lineage composed of ST2059 (*n*=49), ST12347 (*n*=1), and isolates (*n*=7) that belong to five novel sequence types (ST18717, *n*=1; ST18707, *n*=2; ST18727, *n*=1; ST18722, *n*=1; ST18723, *n*=2). These were SLV of ST2059 that displayed recombination within the rhamnose synthesis locus (*rmlACDB*). This resulted in three non-synonymous SNPs (leading to amino acid changes: L214V, R258M, and S272P) in the *rmlA* gene and seven non-synonymous SNPs (leading to amino acid changes: T2S, A12V, E13I, L46E, E57A, D89G, and STOP198E) in the *rmlC* gene.

To evaluate the impact of non-synonymous SNPs in 23F GPSC14 on protein structure, we utilised an AlphaFold model for *S. pneumoniae* Serotype 23F Wzy (UniProt ID: Q9R925), an Alphafold model of RmlA (UniProt ID: A0A2I1UG67) from *Streptococcus oralis* subsp. *dentisani* and an X-ray diffraction model for RmlC (SMTL ID: 1ker.1.) of *Streptococcus suis* [32]. From these three, the I253T mutation in Wzy was predicted to be part of a protein pocket (confidence score of 0.970), and the alteration of the amino acid residue was predicted to induce structural changes in the protein by reducing the cavity volume by 126.576 Å^3^ (Fig. S4). There was no predicted structural changes in the RmlA and RmlC protein models.

**Fig. 3.**
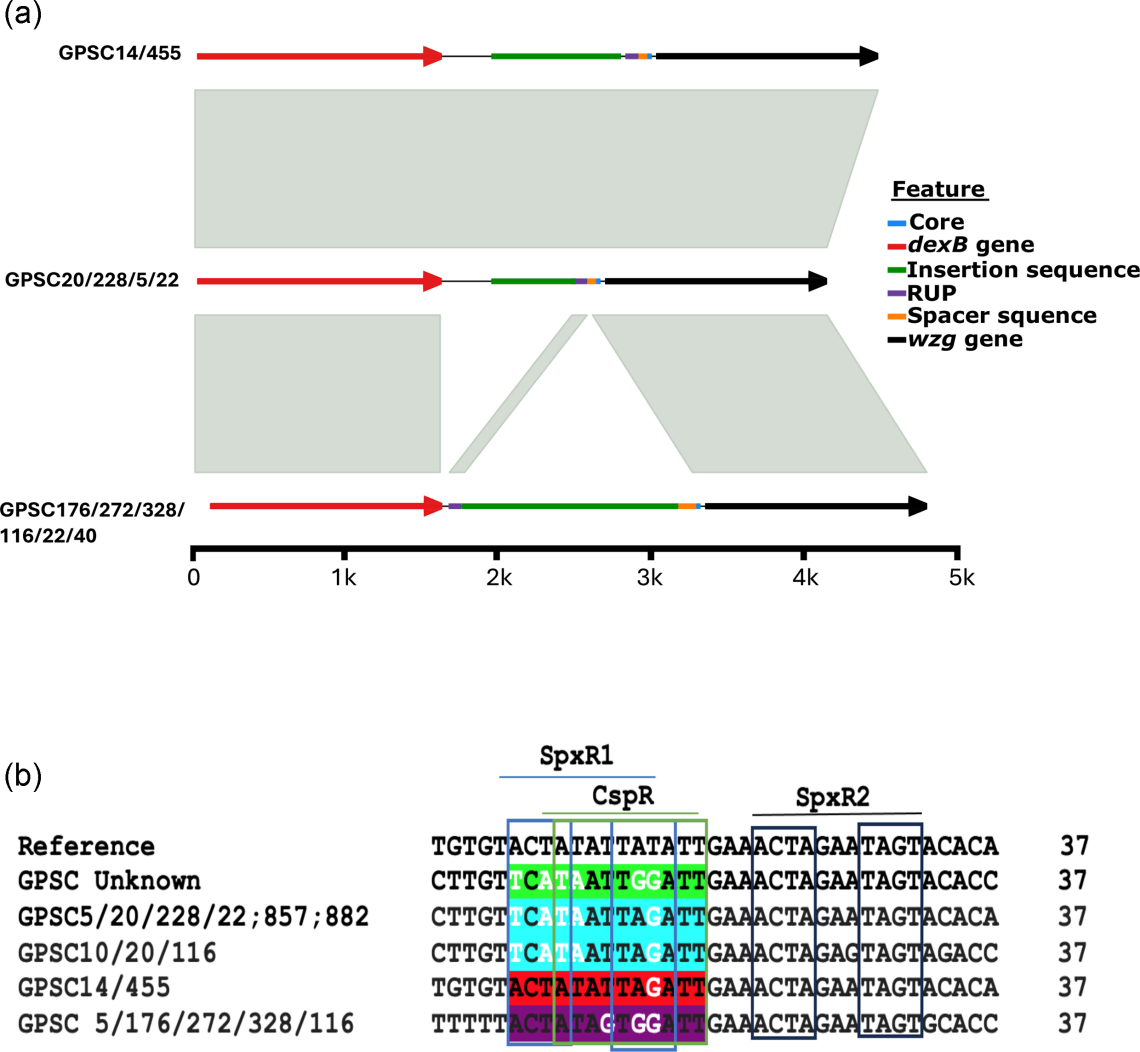
Differences in the *cps* locus intergenic region in the emergent 23F GPSC14 isolates in Blantyre, Malawi. **a**) Genetic synteny of intergenic regions between isolates. The grey box between intergenic sequences of different lineages shows high genetic similarities between them. **b**) Changes in the CpsR and SpxR1 nucleotide-binding sites in the 37-CE. The blue box represents the SpxR1 binding sites, the green box represents the CpsR binding sites, and the black box represents the SpxR2 binding site. Identical sequences within the SpxR1 and CpsR binding regions in 37-CE are indicated by the same colours, and white characters show where there is a SNP in the SpxR1 and CpsR binding regions compared to the reference sequence (D39 Serotype 4).

We then investigated the intergenic regions of the serotype 23F *cps* locus, situated between *dexA* and the *wzg* gene where transcriptional regulators bind to alter capsule expression [27]. We observed structural variations among several different lineages (Fig. 3a). GPSC14 and GPSC455 exhibited a longer intergenic region (1 401 bp) compared to GPSC20, 228, 5, and 22 (554bp) due to the elongation of insertion sequences. Conversely, GPSC176, 272, 328, 116, 22, and 40 displayed a shift in the repeat unit pneumococcal (RUP) preceding the insertion sequences. These structural variations in the intergenic regions of the *cps* locus may lead to different levels of gene expression in the *cps* locus.

Additionally, we identified differences between lineages in DNA binding site sequences for SpxR and CpsR in the 37-CE, which are known to suppress capsule expression [8] (Fig. 3b). Notably, GPSC14 and GPSC455 showed a higher similarity (92 %, SpxR1 and CpsR region) to the reference strain (D39 Serotype 4) in the SpxR1 and CpsR binding sites described by Glanvielle *et al.*, compared to GPSC20, 228, 22, 857, 882, 10, and 116 (62 % similarity, SpxR1 and CpsR region). Moreover, within GPSC5, 176, 272, 328, and 116 there was 72% similarity, SpxR1 and CpsR region compared to the reference sequence. The primary distinction between the GPSC20 SpxR1 and CpsR binding sites and the reference sequence lies in the alterations involving A to T or T to A base changes at positions 1, 3, 4, and 5, along with a T-to-G substitution at position 10. Conversely, for GPSC14 and GPSC455, the sole variation from the reference occurs at position 10, where a T is replaced by a G, whereas GPSC5, 176, 272, 328, and 116 also had two additional changes at positions 7 and 9, both being A to G. Ultimately these changes may alter the binding affinity of the SpxR1 and CpsR proteins, leading to changes in *cps* locus gene expression.

### Emergent serotype 14 isolates with the non-truncated version of the large repetitive protein gene within the *cps* locus

**Fig. 4.**
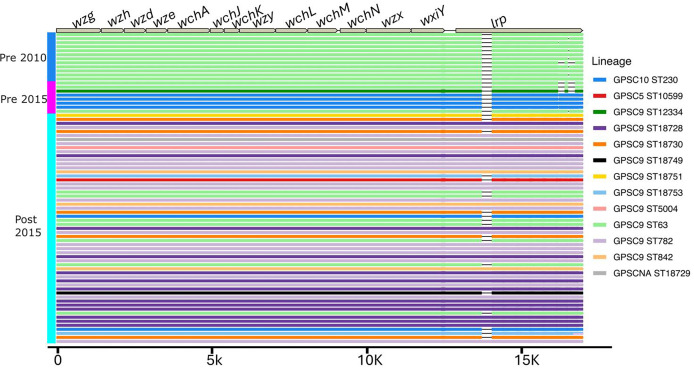
blastn analysis of serotype 14 lineages against the reference serotype 14 sequence to identify large genomic changes in the serotype 14 *cps* sequences, showing the emergence of sequence types within the GPSC9 lineage that have a complete version of the *lrp* gene. Colour blocks on the left represent the time period each isolate was collected. Coloured horizontal lines under the serotype 14 locus represent where there was an alignment between the isolate and the reference sequence (GenBank accession: CR931662), and their colour represents the isolate’s lineage. GPSCNA=non-classified GPSC.

Through a comparative analysis of the serotype 14 *cps* locus over time, we did not observe the emergence of non-synonymous SNPs within *cps* locus genes or changes in *cps* transcriptional binding sites, that we had seen in the 23F lineages. Instead, by blast sequence analysis using a reference serotype 14 sequences (GenBank accession: CR931662) we identified isolates harbouring a large repetitive protein (*lrp*) gene that was not truncated, containing the Cna protein B-type domain a feature absent from serotype 14 isolates *lrp* gene collected before 2015 (Fig. 4). The emergence of isolates with the complete version of the *lrp* gene post-PCV13 introduction may suggest that the bacteria have a fitness advantage, potentially aiding them in evading vaccine-induced antibodies through alterations in the bacteria’s immunogenicity.

### Global comparative analysis of serotype 23F Wzy, SpxR1 and CpsR binding motif, and serotype 14 Lrp changes

To further understand whether the mutations related to the serotype 23F Wzy amino acid structure and the SpxR1 and CpsR binding sites in 37-CE are specific to certain lineages, and if they are confined to Malawi, we conducted genomic comparisons with 1290 serotype 23F isolates that were collected worldwide from the pathogenwatch database including isolates from Malawi from different regions (Table S2). Notably, mutations in Wzy protein sequences and the genetic structures of the 37-CE were consistent across the majority of GPSC14 isolates globally. Moreover, the Wzy protein in serotype 23F exhibits a high level of sequence conservation across all serotype 23F isolates, there were only ten distinct versions of the protein but only two that were found in more than one isolate within the available dataset. The emergence of a mutated Wzy protein in GPSC14 isolates suggest it may provide the bacteria a fitness benefit. Also, different versions of the 37-CE sequence are primarily associated with specific lineages which implies that different lineages may be linked to varying levels of *cps* expression.

In our core-genome maximum likelihood phylogenetic analysis of serotype 23F GPSC14 isolates, constructed by aligning against the complete genome of GPSC14 *S. pneumoniae* (GenBank accession: NZ_LR216043), we find close genetic relationships between the newly emergent GPSC14 ST2059 in Blantyre and GPSC14 ST2059 isolates from South Africa and Mozambique, with the closest South African isolate differing by only seven SNPs and Mozambique isolate by eight SNPs (Fig. S5). This implies possible country-to-country transmission, most likely from South Africa to Malawi, as the ST2059 lineage was prominent in South Africa pre-and post-PCV vaccine introduction but not in Malawi.

We also conducted a comparative analysis of serotype 14 isolates' *lrp* gene. Utilising data from 1607 isolates collected globally, including Malawian isolates from various regions, our aim was to determine whether the non-truncated version of the *lrp* gene is associated with specific lineages (Table S3). Interestingly, we observed that, aside from GPSC9, other lineages mainly had either the complete or truncated version of the gene, indicating that the versions of the gene evolved separately from each other (Table S4). Our phylogenetic analysis of GPSC9 serotype 14 isolates, aligned against the serotype 14 GPSC9 *S. pneumoniae* complete genome (GenBank accession: CP001015), indicates a genetic divergence based on the completeness of the *lrp* gene (Fig. S6). We observed one cluster containing all truncated versions of the *lrp* gene, and the other comprised a mixture of complete and incomplete *lrp* genes, suggesting that variations in the *lrp* gene could potentially play a role in the differences observed among these isolates.

### Emergent serotype 23F and 14 lineages are associated with virulence genes and AMR

To explore the hypothesis that the emerging serotype 23F and 14 lineages had additional virulence and AMR characteristics that conveyed advantage, we first compared the accessory gene content of the different lineages [26] (Table 1). A toxin-antitoxin system gene, which is associated with bacterial stress response, cell growth, and biofilm formation, is present in the Serotype 23F GPSC14 isolates [51,52]. Additionally, the pneumococcal serine-rich repeat protein (PsrP)-accessory Sec system (secY2A2) pathogenicity island, involved in biofilm formation, adhesion to epithelial cells, and the export of the contact-dependent pneumolysin toxin, were also consistently found in these isolates [53,55]. However, both these genetic elements are absent in the 23F GPSC20 isolates. Furthermore, all 23F ST2059 isolates carried the macrolide resistance gene *mefA*, which was only present in three 23F isolates outside of GPSC14. Additionally, they harboured Thiazolylpeptide-type bacteriocin and Lantibiotic resistance genes associated with bacterial colonisation [56].

**Table 1.** Selected accessory virulence genes present in the emergent serotype 23F and 14 lineages

Lineage/Strain	Gene function	Sensitivity	Specificity
Serotype 23F GPSC14	Toxin-antitoxin system toxin component domain protein; CI-like repressor metallo-proteinase motif protein; Imm40 domain-containing protein; ImmA/IrrE family metallo-endopeptidase	100.0	93.4
	Accessory Sec system protein Asp2	100.0	78.7
	Accessory Sec system protein translocase subunit SecY2	100.0	78.7
	Accessory Sec system protein Asp1	100.0	78.7
	Accessory Sec system protein Asp2	100.0	78.7
	Accessory Sec system protein translocase subunit SecY2	100.0	78.7
	Glycosyltransferase GlyF; Glycosyl transferase family 8	100.0	78.7
	Glycosyl transferase family 2; Glycosyltransferase GlyG	100.0	78.7
	Accessory Sec system protein Asp3	100.0	78.7
	Glycosyltransferase GlyE	100.0	78.7
	Sugar transferase gtf3	100.0	78.7
	Accessory Sec system translocase SecA2	100.0	77.0
	Accessory Sec system glycosylation chaperone GtfB	100.0	77.0
	Accessory Sec system glycosyltransferase GtfA	100.0	77.0
Serotype 23F GPSC14 ST2059	Macrolide efflux MFS transporter Mef(A)	100.0	76.6
	ABC-F type ribosomal protection protein Msr(D)	100.0	76.6
	Lantibiotic biosynthesis protein	100.0	72.7
	Lantibiotic efflux protein	100.0	72.7
	Lantibiotic biosynthesis protein	100.0	72.7
	Thiazolylpeptide-type bacteriocin	100.0	72.7
Serotype 14 GPSC9 cluster C	Choline binding protein PcpA	97.0	97.3
Serotype 14 GPSC ST18728	Zinc metalloprotease ZmpB	100.0	98.2

**Fig. 5.**
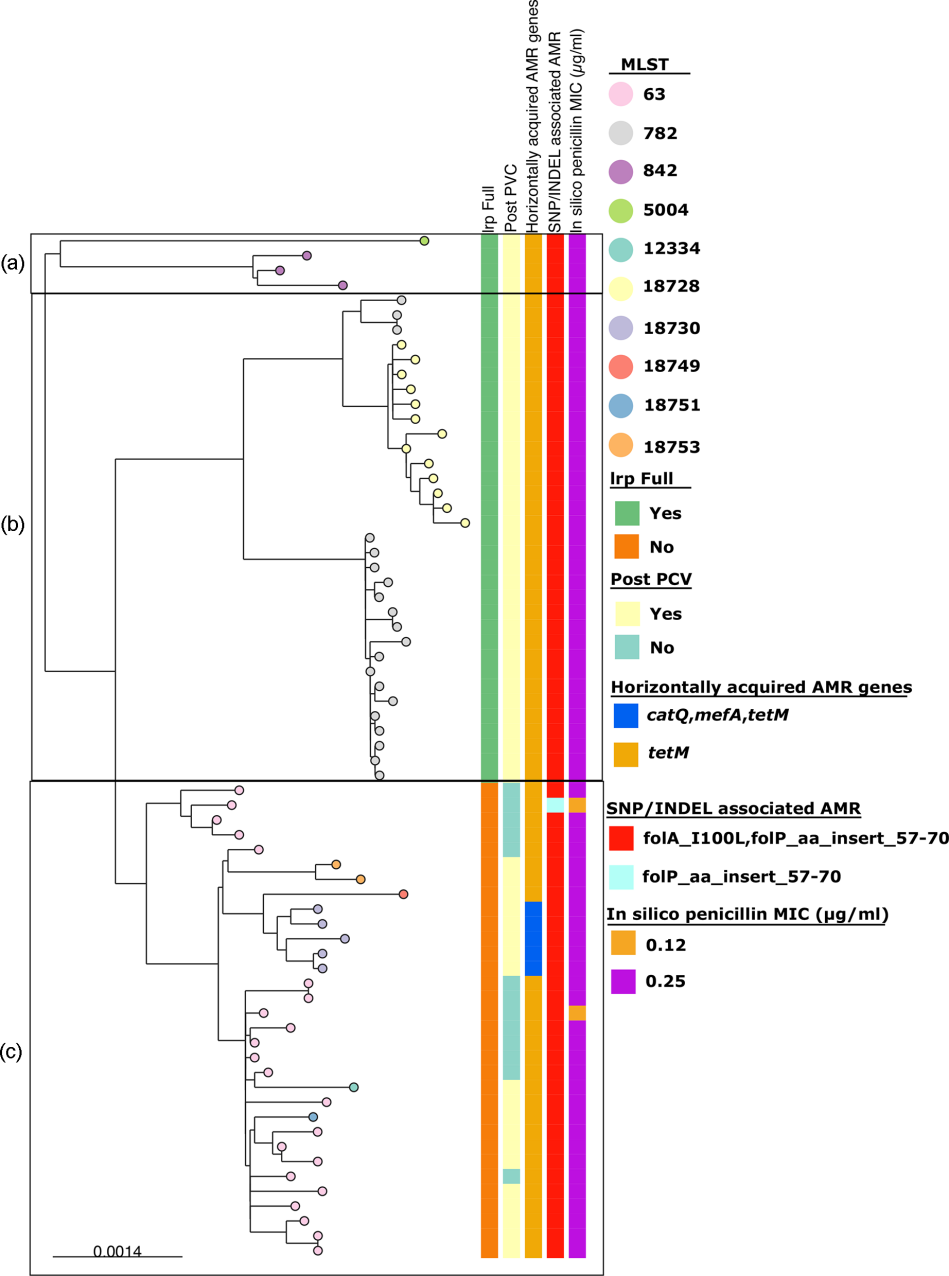
Core-genome phylogenetic tree of serotype 14 GPSC9 isolates from Blantyre, Malawi, and the clustering used to define different sub-lineages of GPSC9 for accessory gene content comparison. The right side of the figure shows the phylogenetic tree with tips of the tree representing their MLST, and the left side features colour tiles representing isolates by their version of the *lrp* gene, isolated pre- or post-PCV13 introduction, and their horizontally acquired AMR genes, SNP/INDEL-associated AMR, and the *in silico* penicillin MIC value. The box around parts of the tree and letters (**a–c**) represent the sub-lineage clusters used to compare the accessory gene content in Table 1.

Regarding serotype 14 GPSC9 lineage, we divided them into three distinct phylogenetic clusters (A, B, and C) to determine the differences in the accessory genome among these clusters with cluster C containing PCV13 emergent lineages (Fig. 5). The choline-binding protein *pcpA* virulence gene which mediates pneumococcal adhesion was present in all but one isolate in cluster C [57]. ST18728 also carried the zinc metalloprotease virulence gene (*zmpB*) associated with inflammation in the lower respiratory tract, found in only one additional isolate indicating the emergent isolates may have an increased virulence potential enabling the lineage to the dominate lineages pre-PCV13 introduction [58].

**Fig. 6.**
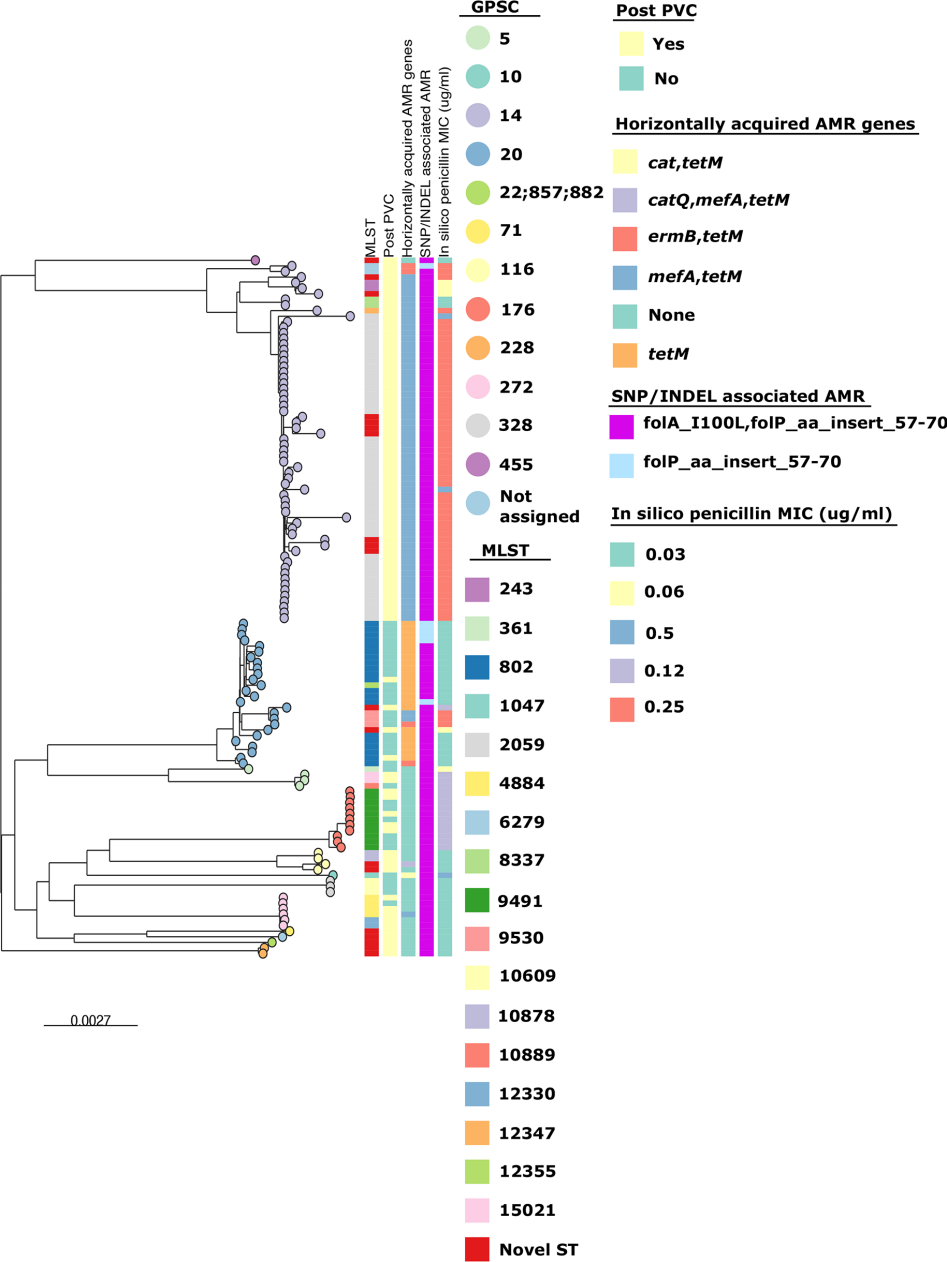
Core-genome phylogenetic tree without recombination removal of serotype 23F isolates from Blantyre, Malawi showing the emergent GPSC14 ST2059 isolates having resistance to more antibiotic classes from genotype data over other lineages. Right side of figure is the phylogenetic tree with tips of the tree representing their MLST and left side are colour tiles represent isolates MLST, pre- or post-PCV13 introduction, horizontally acquired AMR genes, SNP/INDEL associated AMR and the *in silico* penicillin MIC value.

To assess AMR among the emergent serotype 23F and 14 lineages, we compared them to the lineage that dominated before PCV13 introduction (Fig. 6). For 23F ST2059 harboured *mefA* (macrolide resistance), *tetM* (tetracycline resistance) and the mutations folP_aa_insert_57–70 and folA_I100L (resistance to sulfamethoxazole and trimethoprim, respectively). Additionally, 23F ST2059 isolates were penicillin non-susceptible (MIC 0.25 to 0.5 µg µl^−1^), unlike GPSC20 ST82, which were penicillin-susceptible (MIC 0.035 µg µl^−1^) (Fig. 5). There were three 23F GPSC20 ST9530 isolates recovered before PCV13’s introduction which also exhibited penicillin non-susceptibility (MIC 0.25 µg µl^−1^).

For serotype 14 isolates there were no additional gene mutations (folP_aa_insert_57_70 and folA_I100L) or acquisitions (*tetM*) conferring AMR. All serotype 14 lineages were penicillin non-susceptible. There was no additional increase in penicillin MIC amongst the emerging GPSC9 lineage.

Sensitivity is the presence of this gene within the target genotype and specificity is the absence of this gene within the non-target genotype.

## Discussion

In the context of persistent *S. pneumoniae* VT carriage and high PCV13 uptake in Malawi, we have identified serotypes 23F and 14 lineages, serotypes that have been prominent causes of IPD, with potentially functionally important genetic changes in their *cps* locus [13,59]. These lineages harbour additional virulence attributes and AMR, which may further enhance their potential for vaccine escape. Indeed, together with a recently described serotype 3 GPSC10- ST700, clonal expansion of these lineages may explain the residual circulation of some VT serotypes after vaccination in Blantyre, Malawi [13]. The detection of these lineages was only possible through continuous genomic surveillance at the population level, highlighting the importance of such surveys following PCV introduction.

The predominant serotype 23F lineage before PCV13 introduction in Malawi was GPSC20 ST802, a lineage found on multiple continents [60,61]. However, post-PCV13 introduction, there was a decrease in ST802, and an expansion of GPSC14 ST2059, which, to date, has only been reported from neighbouring South Africa and Mozambique [60,62]. Intriguingly, data from South Africa suggests that 23F ST2059 has a more invasive phenotype [27]. Whether vaccine pressure was directly responsible for this shift is uncertain, as genotype replacement of 23F isolates has been reported in China (GPSC24 ST342 to multidrug-resistant GPSC16 ST81 also known as the PMEN1 clone lineage) which does not have a routine PCV programme [61]. However, once established in Malawi, the genotypic attributes of the 23F GPSC14 ST2059 lineage may explain its persistence, particularly in the context of vaccine-induced serotype-specific immunity that has waned below the correlates of protection for both carriage and disease in the first year of life [14].

We have identified two mutations within the *cps* locus of the emergent 23F GPSC14 lineage that were absent in other 23F genetic lineages. These are predicted to have an impact on capsule expression and production [36,63]. These mutations included *wzy*, an oligosaccharide repeat unit polymerase responsible for linking sugar chains outside the cell wall as part of the capsule. Wzy shows significant diversity between serotypes but is highly conserved within serotypes [20,64]. It was therefore unexpected to find that the 23F GPSC14 lineage has multiple non-synonymous SNPs, including a SNP I253T that reduces the cavity volume found within a protein pocket of Wzy. Mutagenesis studies of Wzy involved in lipopolysaccharide synthesis in Gram-negative bacteria such as *Pseudomonas aeruginosa* and *Shigella flexneri*, affect the O-antigen chain length and distribution affecting capsule thickness [65,66]. Moreover, differences in the intergenic region structure, especially the SNPs in the SpxR1 and CpsR DNA binding sites in the 37-CE have previously been shown to alter the level of capsule production affecting the strain’s virulence [36,63]. Furthermore, recent phenotypic studies have revealed a 6.6-fold variation in cps production among 23F isolates, though the lineage and *cps* locus genotype was not explored [67]. We therefore hypothesise that mutations in *wzy* and 37-CE will most likely affect capsule function and expression respectively, conferring a competitive advantage.

The prevalent serotype 14 lineage before PCV13 introduction in Malawi was GPSC9 ST63, a globally distributed sequence type [68]. Post-PCV introduction, *S. pneumoniae* GPSC9 isolates from the USA, Israel, South Africa, and Cambodia have been identified that have switched serotype from 14 to nonvaccine type 15A [68]. This has not been seen in Malawi, instead GPSC9 ST63 has been overtaken by an expanding serotype 14 GPSC9 ST782, another global sequence type [69,70]. Moreover, there was also the expansion of the phylogenetically closely related novel sequence type, ST18728, which to date has only been reported in Malawi. This expanding serotype 14 GPSC9 strain is associated with a genetic alteration in the *lrp* gene, where we identified the emergent strain GPSC9 ST782 and a novel sequence type closely related to Blantyre’s ST782, which harbours the complete 1359-amino acid-long version of the Lrp protein containing the Cna protein B-type collagen-binding domain. Variants in the *lrp* gene have been previously reported, particularly the serotype 14-like isolates from Papua New Guinea [71,72]. However, the Papua New Guinea isolates differed from Blantyre isolates, not reacting to serotype 14 antibodies, and lacking key genes. The Lrp protein’s function is unknown, but it’s hypothesised to be a dominant antigen, overriding serological similarities between serotype 14 and 15 [8]. The Lrp protein’s collagen-binding Cna B domain, also found in the virulence factor RrgB protein, could affect serotype 14’s colonisation ability and immunogenicity [67,68].

In addition to changes in the *cps* locus, possible factors contributing to the emergence and spread of these 23F and 14 lineages include genes associated with AMR, colonisation, and virulence that would provide bacteria with an advantage over lineages from the same setting. The question is whether these lineages that seem to have a competitive advantage will disseminate more widely. It is noteworthy that a different serotype 23F lineage, PMEN1, identified before pneumococcal vaccines were widely introduced, became transmitted across the globe, causing invasive disease [73,75]. The emergence and spread of PMEN1 was attributed to the acquisition of antibiotic resistance, transmission, and virulence genes absent in closely related ancestor strains [76]. Together this shows the importance of identifying emergent strains with genetic adaption and assessing their potential to transmit locally and globally.

The main limitation of this study is the limited number of genomes from Malawi prior to and immediately after the introduction of PCV13 and that these historical genomes were biassed towards invasive rather than carriage isolates. However, we speculate that, based on closely related isolates from neighbouring countries, such as the ST2059 isolates in South Africa, that these expanding lineages are likely to cause invasive disease in Malawi [77,78].

In conclusion, following the introduction of PCV13 in Malawi, there has been clonal expansion of 23F and 14 lineages, characterised by genotypic *cps* locus changes that affect capsule expression and production, and potentially interaction with the host immune system. These lineages also have genetic features that confer a competitive advantage in terms of colonisation, transmission and AMR. These findings underscore the value of robust *S. pneumoniae* genomic surveillance to inform vaccine programmes in high burden settings where, in the face of a high force of infection, sufficient control of pneumococcal colonisation and disease has not yet been achieved [11,79, 80].

## supplementary material

10.1099/mgen.0.001264 Fig. S1

10.1099/mgen.0.001264 Table S1

## Abbreviations

CPS: capsule polysaccharide
GPS: Global Pneumococcal Sequencing Project
GPSC: Global Pneumococcal Sequence Cluster
IPD: invasive pneumococcal disease
Lrp: large repative protein
MLST: multilocus sequence typing
NVT: non-vaccine serotypes
PCV: pneumococcal conjugate vaccine
PCV13: 13-valent pneumococcal conjugate vaccine
PCVPA: pneumococcal conjugate vaccine prospective analysis
VT: vaccine type serotype
