# Supplementary material for: Expansion of pneumococcal serotype 23F and 14 lineages with genotypic changes in capsule polysaccharide locus and virulence gene profiles post introduction of pneumococcal conjugate vaccine in Blantyre, Malawi
Source: Microb Genom. 2024 Jun 19;10(6):001264. doi: 10.1099/mgen.0.001264 (PMC11261835; doi:10.1099/mgen.0.001264)
Supplement: Fig. S1 [file mgen-10-01264-s001.pdf]

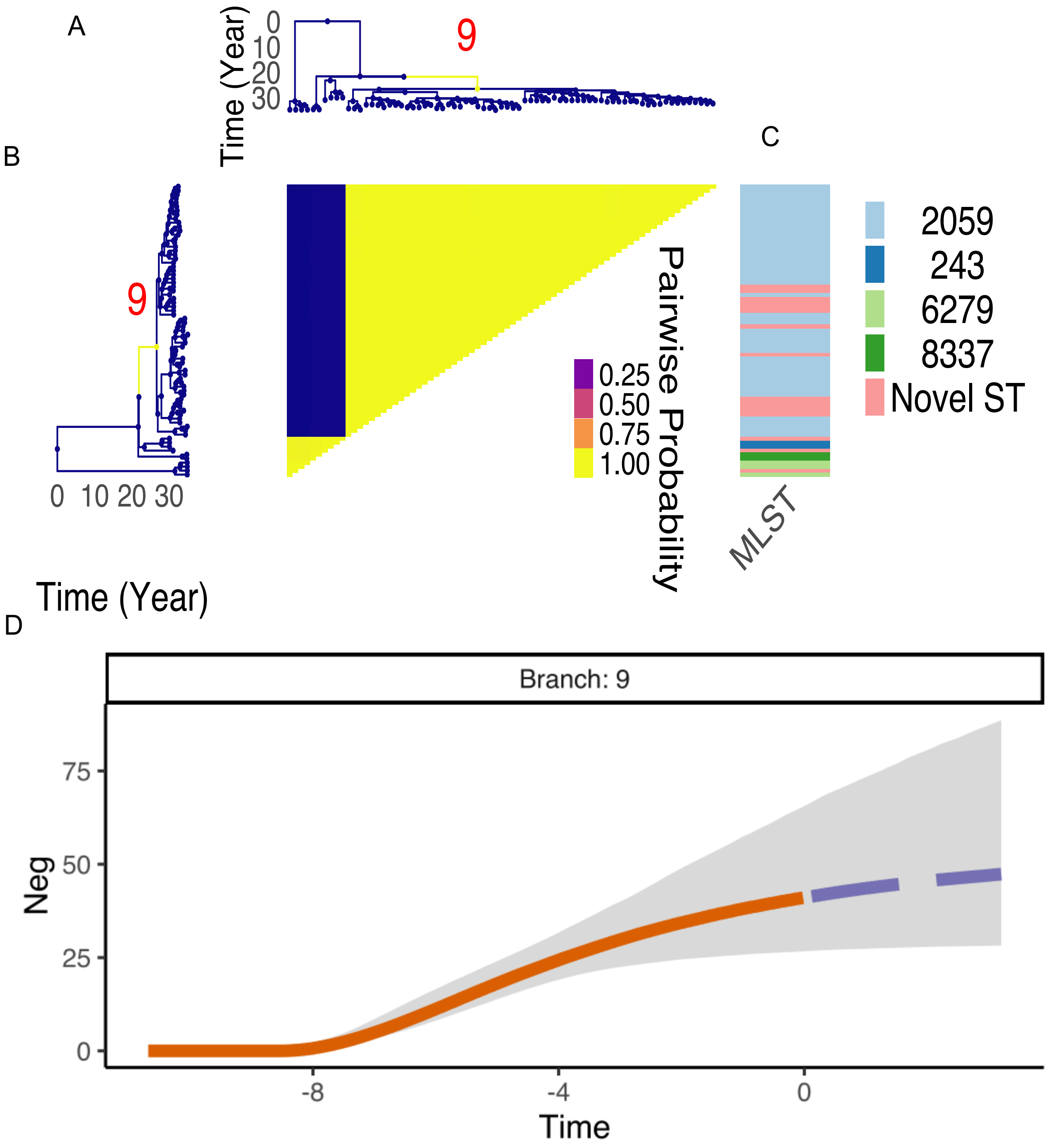

Fig. S1: Expansion and inferred effective population size of GPSC14 ST059 isolates in Blantyre, Malawi . A) Dated phylogeny with branch coloured according to the probability of clonal expansion. B) Pairwise matrix showing the posterior probabilities of any two genomes belonging to the same subpopulation. C) Colour tile showing MLST of each isolate. D) Posterior summary of the inferred effective population size function. Grey area 95% credible interval and the lines represent median. Solid denotes past effective population size inference and dashes represents prediction of future effective population size. Point 0 on x-axis represent most recent sample date which was 2019.

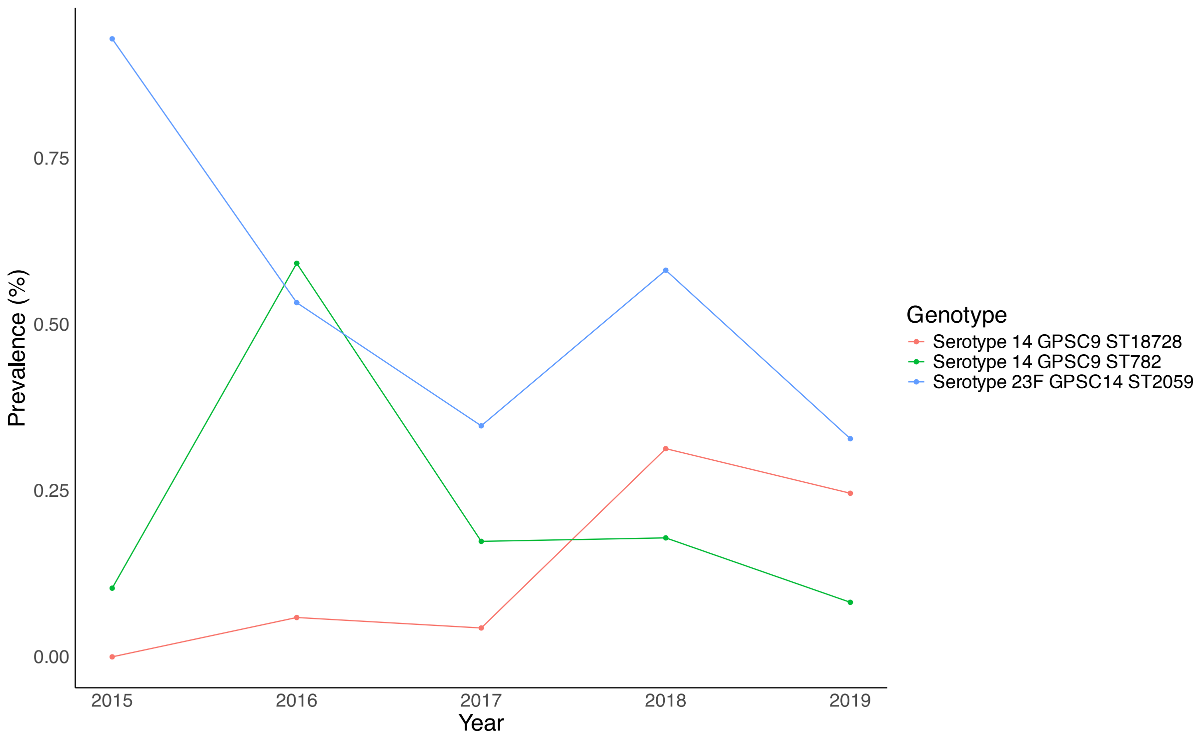

Fig S2: Prevalence of serotype 23F GPSC14 ST2059, Serotype 14 GPSC9 ST782 , Serotype 14 GPSC9 ST18728 lineages over time in the PCVPA dataset.

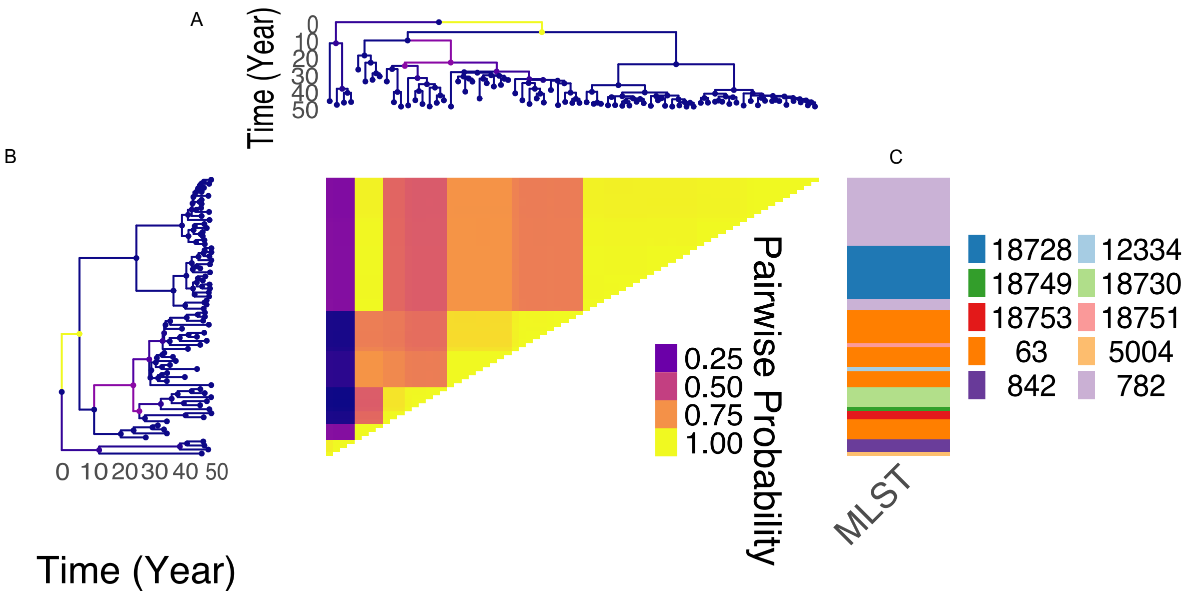

Fig. S3: Predicted expansion within GPSC9 lineage isolates in Blantyre, Malawi. A) Dated phylogeny with branch according to the probability of clonal expansion. B) Pairwise matrix showing the posterior probabilities of any two genomes belonging to the same subpopulation. C) Colour tiles showing MLST of each isolate.

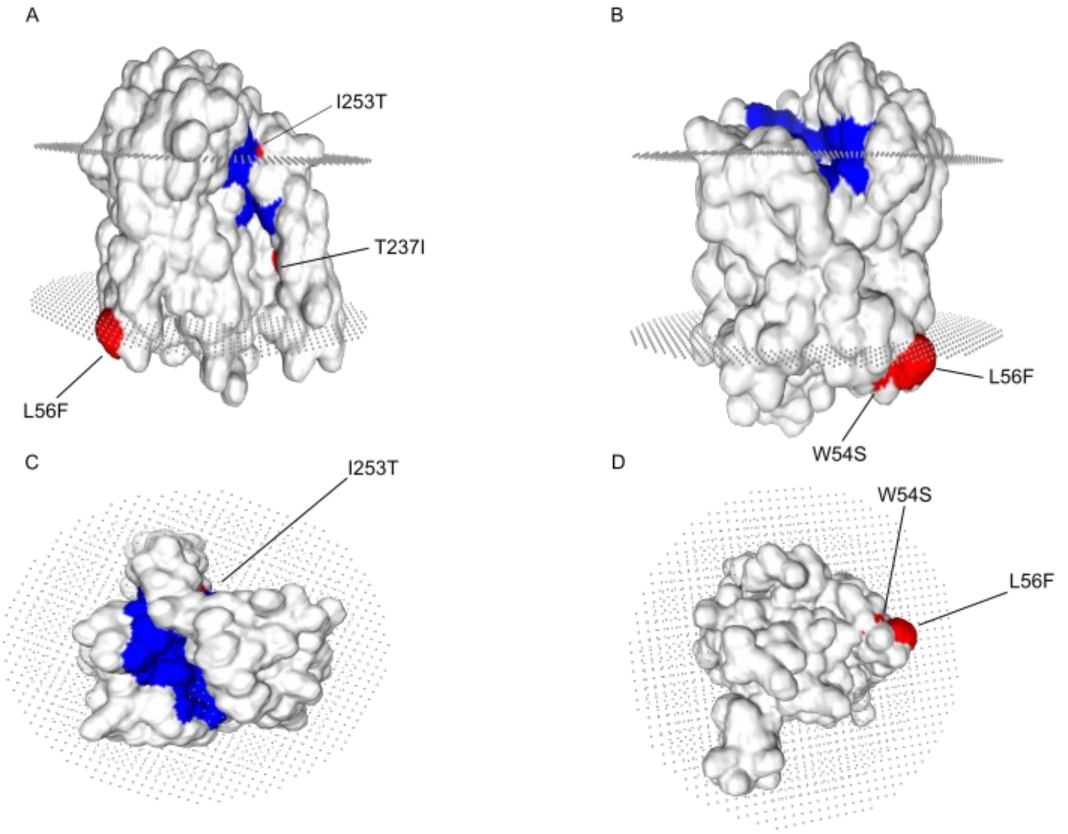

Fig. S4: 3D Structure of Serotype 23F Wzy protein from an AlphaFold Model (PDB accession: Q9R925P), showing the I253T mutation which causes a structural change in the protein, found within a protein pocket. A) Front view B) back View C) Extracellular view D) Cytoplasmic view. Grey dots represent the bacterial membrane. Blue-highlighted amino acid residues represent the predicted protein cavity pocket, while red-highlighted amino acid residues represent amino acid changes from non-synonymous mutation.

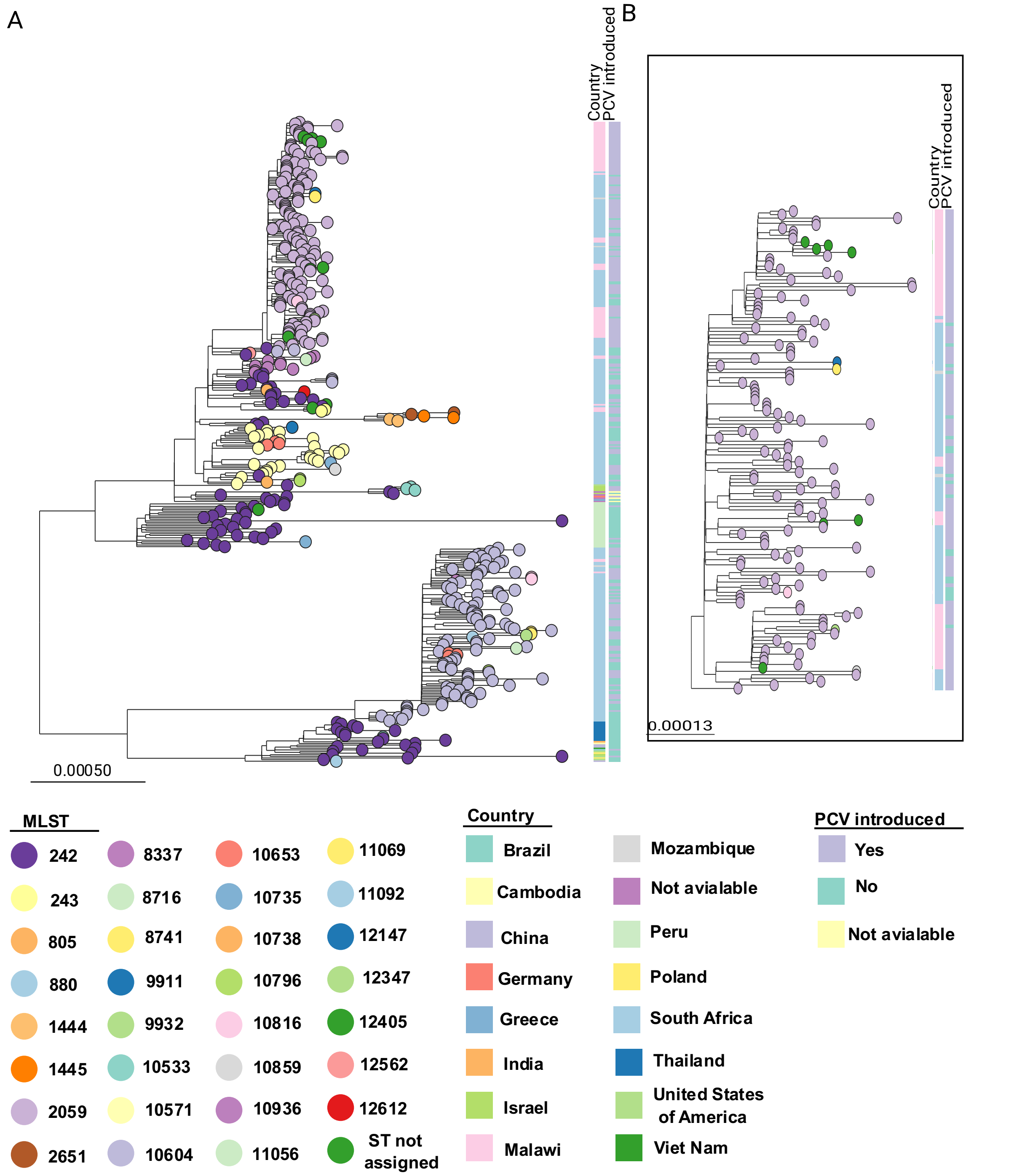

Fig. S5: Serotype 23F GPSC 14 core-genome SNP Phylogenetic tree showing closely genetically relationship between Malawian, South African and Mozambique ST059 isolates. A) Whole phylogenetic tree of GPSC14 cluster B) Zoom into the ST2059 cluster on the GPSC14 phylogenetic tree. Left side of figure A and B is the phylogenetic tree is the with tips representing their MLST. Right side of figure A and B is colour tiles representing the country isolates from and if they were isolated pre or post PCV13 introduction.

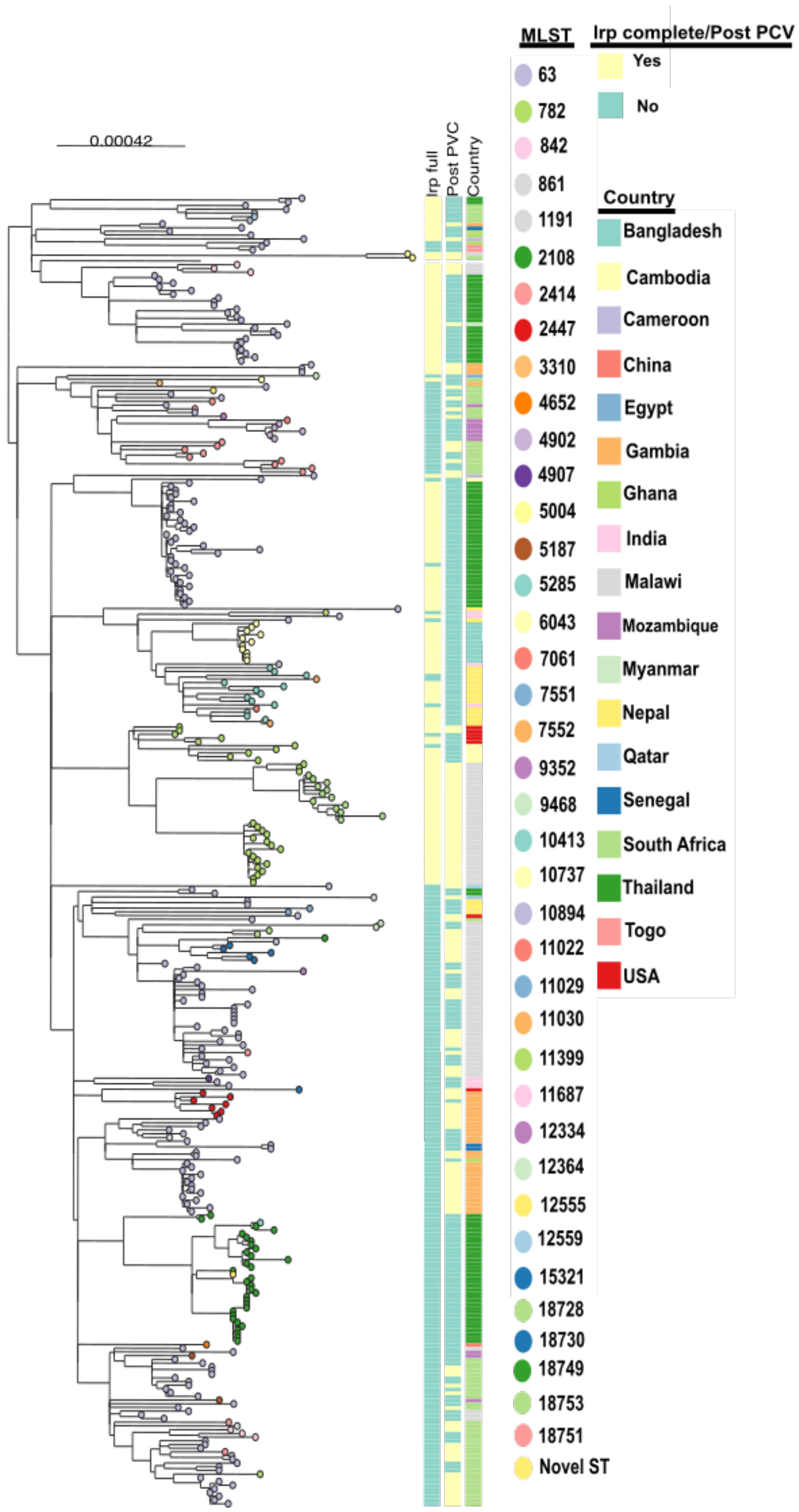

Fig. S6: Serotype 14 GPSC9 core SNP phylogenetic tree showing the divergence in lineages related to having complete or truncated versions of the lrp gene. The left side of the figure is the phylogenetic tree with tips representing their MLST. The right side is a colour tile

representing isolates' lrp gene versions, whether they were isolated pre- or post-PCV13 introduction, and the country from which they were isolated.

Table S4: Distribution of complete and truncated versions of serotype 14 *lrp* gene across genetic lineages

	Complete *lrp* gene	
GPSC	Yes(%)	No (%)
18	489 (98.39)	8 (1.61)
39	159 (100)	0 (0)
904;9	149 (40.93)	215 (59.07)
Not assigned	2 (66.67)	1 (33.34)
5	0 (0)	1 (100)
10	1 (0.62)	158 (99.37)
6	345 (99.71)	1 (0.28)
16	16 (100)	0 (0)
288	5 (100)	0 (0)
108	28 (96.55)	1 (3.45)
279	5 (100)	0 (0)
14	1 (50)	1 (50)
4	7 (100)	0 (0)
1	1 (100)	0 (0)
90	0 (1)	1 (100)
301	0 (0)	4 (100)
238	4 (100)	0 (0)
571	1 (100)	0 (0)
103	1 (100)	0 (0)
28	0 (0)	71 (100)
79	1 (100)	0 (0)
21	0 (0)	1 (100)
27	1 (100)	0 (0)
11	1 (100)	0 (0)
